# Identification of cellular microRNA miR-188-3p with broad-spectrum anti-influenza A virus activity

**DOI:** 10.1186/s12985-020-1283-9

**Published:** 2020-01-30

**Authors:** Huan Cui, Chunmao Zhang, Zongzheng Zhao, Cheng Zhang, Yingying Fu, Jiaming Li, Guanxi Chen, Mengxi Lai, Zhixiang Li, Shishan Dong, Ligong Chen, Zhaoyang Li, Chengyu Wang, Juxiang Liu, Yuwei Gao, Zhendong Guo

**Affiliations:** 1 Institute of Military Veterinary, Academy of Military Medical Sciences, 666 West Liuying Road, Changchun, 130122 Jilin China; 2 College of Veterinary Medicine, Hebei Agricultural University, 2596 Lucky South Street, Baoding, 071000 Hebei China; 3 School of Life Science and Engineering, Southwest University of Science and Technology, Mianyang, 621010 Sichuan China; 4 Department of Emergency, Baoding First Central Hospital, Baoding, 071000 Hebei China

**Keywords:** Influenza A virus, miRNA, Broad-spectrum, Antiviral activity

## Abstract

**Background:**

Influenza A virus (IAV) continues to pose serious threats to public health. The current prophylaxis and therapeutic interventions for IAV requires frequent changes due to the continuous antigenic drift and antigenic shift of IAV. Emerging evidence indicates that the host microRNAs (miRNAs) play critical roles in intricate host-pathogen interaction networks. Cellular miRNAs may directly target virus to inhibit its infection and be developed as potential anti-virus drugs.

**Methods:**

In this study, we established a broad-spectrum anti-IAV miRNA screening method using miRanda software. The screened miRNAs were further verified by luciferase assay, viral protein expression assay and virus replication assay.

**Results:**

Five cellular miRNAs (miR-188-3p, miR-345-5p, miR-3183, miR-15-3p and miR-769-3p), targeting 99.96, 95.31, 92.9, 94.58 and 97.24% of human IAV strains recorded in NCBI, respectively, were chosen for further experimental verification. Finally, we found that miR-188-3p downregulated PB2 expression at both mRNA and protein levels by directly targeted the predicted sites on *PB2* and effectively inhibited the replication of IAV (H1N1, H5N6 and H7N9) in A549 cells.

**Conclusions:**

This is the first report screening cellular miRNAs that broad-spectrum inhibiting IAV infection. These findings suggested that cellular miR-188-3p could be used for RNAi-mediated anti-IAV therapeutic strategies.

## Background

Influenza A virus (IAV) is a kind of single negative-stranded RNA virus that belongs to the Orthomyxoviridae family [1]. It is the causative agents for both seasonal influenza and pandemic influenza, posing major public health challenges [2, 3]. The annual epidemics of seasonal influenza caused 3–5 million cases of severe illness worldwide. In addition, four influenza pandemics have been recorded since the twentieth century: the 1918 Spanish flu, the 1957 Asian flu, the 1968 Hong Kong flu and the 2009 swine flu [4, 5], leading to an estimated 300,000 to 50 million deaths worldwide [6, 7]. Recently, parts of highly pathogenic avian IAVs acquired the ability to cross the interspecies barrier causing sporadic infections in humans with high fatality rate, such as H5N1 [8], H5N6 [9] and H7N9 [10]. Vaccination and small-molecule antiviral drugs (such as M2 ion channel blockers and neuraminidase inhibitors) are considered the best options for control of influenza infection [11]. However, because of the easily occurrence of antigenic drift and antigenic shift, influenza vaccines need to be updated annually and the number of reports of drug-resistant influenza strains keeps increasing [12, 13]. Particularly, more than 95% of the current circulating IAV strains are resistant to M2 ion channel blockers [14]. The continued threat of epidemic and pandemic outbreaks and the limitations of current antiviral strategies underscore the urgent need for developing new influenza therapies.

MicroRNAs (miRNAs) are a class of ~ 22 nucleotides (nt) small regulatory non-coding RNA that are conserved expressed by animals, plants and viruses [15, 16]. They were reported to play a pivotal role in gene regulation by repressing or degrading target mRNA [17] and participate in various cellular process, including cell growth, differentiation, apoptosis, homeostasis, and tumorigenesis [18–22]. Recently, it has been found that miRNAs also implicated in the regulation of virus invasion [23]. Lecellier et al. [24] reported that miR-32 effectively restricted the accumulation of the retrovirus primate foamy virus type 1 (PFV-1) in human cells. Huang et al. [25] reported that the 3′ ends of HIV-1 messenger RNAs were targeted by a cluster of cellular miRNAs including miR-28, miR-125b, miR-150, miR-223 and miR-382, contributing to HIV-1 latency. Song et al. [26] reported that miR-323, miR-491, and miR-654 inhibit replication of the H1N1 influenza A virus through binding to the same conserved region of the PB1 gene. Let-7c [27] was found to regulate influenza virus replication through the degradation of viral gene (+) cDNA by matching the 3’UTR of the M1(+) RNA. Zhang et al. [28] reported that *Sus scrofa* miR-204 and miR-4331 negatively regulate swine H1N1/2009 IAV replication by targeting viral HA and NS, respectively. miR-127-3p, miR-486-5p and miR-593-5p were found to target at least one viral gene segment of both the human seasonal influenza H3N2 and PR8 (H1N1) virus [29]. miR-122 [30] is essential for hepatitis C virus replication in liver, and Lanford et al. [31] found that treatment of chronically infected chimpanzees with anti-miR-122 leads to long-lasting suppression of HCV viremia, with no evidence of viral resistance or side effects in the treated animals.

In summary, some cellular miRNAs may have direct antiviral effects in addition to its known cellular functions, indicating that miRNAs can be developed as a new effective therapeutic strategy to subdue viral infections. However, the broad-spectrum antiviral property of miRNAs had not been studied before. Here, we developed a broad-spectrum antiviral miRNA screening strategy to screen cellular miRNAs that both effectively and universally inhibited the replication of IAV. miRanda software was used to predict the potentially bindings between all human mature miRNAs (2656 records) and all human IAV strains (28,124 records). Five cellular miRNAs that universally target PB1, PB2, PA or NP gene of IAV were selected. To determine the antiviral effectiveness of these miRNAs, the performance of inhibiting target viral protein expression and virus replication was evaluated. Finally, we found miR-188-3p, potentially targeting 99.96% of human IAVs, could effectively repress IAV (H1N1, H5N6 and H7N9) replication in infected A549 cells by targeting PB2 mRNA, suggesting that cellular miR-188-3p may be a potential therapeutic strategy to inhibit IAV infection.

## Materials and methods

### Cells and viruses

The human renal epithelial cells (HEK-293 T) and Madin-Darby canine kidney cells (MDCK) were purchased from the American Type Culture Collection (ATCC) and cultured in Dulbecco’s Modified Eagle Medium (DMEM) with 10% fetal bovine serum (FBS), 100 U/ml penicillin and 0.1 mg/ml streptomycin. Human lung epithelial cells (A549) were purchased from ATCC and maintained in RPMI 1640 media supplemented with 10% FBS, 100 U/ml penicillin and 0.1 mg/ml streptomycin. All cells were cultured at 37 °C in a 5% CO2 incubator with humidified air. Influenza A viruses, A/FM/1/47(H1N1) (abbreviated as FM47), A/quail/Hebei/CH06–07/2018(H7N9) (abbreviated as QA07) and A/chicken/Hubei/XY918/2016(H5N6) (abbreviated as CK918), were propagated in 9-day-old embryonated chicken eggs (Specific Pathogen Free, Merial-Vital Laboratory Animal Technology, Beijing, China) for 48–72 h at 35 °C. The allantoic fluid was clarified by centrifugation at 3,000 rpm, 4 °C for 10 min and stored at − 80 °C until use. Virus production was titrated in MDCK cells and titers were calculated by the method developed by Reed and Muench. This study was approved by the Biosafety Committee and Ethics Committee of the Institute of Military Veterinary.

### Bioinformatic analysis

Sequence of Influenza A virus was downloaded from NCBI influenza virus Resource (http://www.ncbi.nlm.nih.gov/genomes/FLU/FLU.html). The sequence of strains whose host was human and all eight segments had full-length was extracted for further analysis. Computer program miRanda software 3.3a [32, 33] was used to scan the genomes of human Influenza A virus for the presence of target sites for the human miRNAs listed in miRbase (http://www.mirbase.org/). The cutoff values for miRanda score and minimal free energy of binding were set to 140 and − 15 kcal/mol. An exact matching to 5′ end seed region (positions 2–8) of the mature miRNA was used and the G:U base pairing was not allowed. Other parameters of the software were kept as default. miRNA-target gene pairs were confirmed using RNAHybrid at http://bibiserv.techfak.uni-bielefeld.de/.

### Plasmid construction

3′-UTR reporter analysis experiments were used to assess the potential miRNA targets on Influenza A virus. Fragments that containing potential miRNA target were amplified by PCR and directly cloned into pGL3-cm, in which the multiple cloning site of the pGL3-control vector (Promega, Madison, WI, USA) was removed and placed downstream of the luciferase gene as described previously [34]. These constructed vectors were named pGL3-PB2–188-3p, pGL3-PB2–345-5p, pGL3-PB1–3183, pGL3-PA-15a-3p, and pGL3-NP-769-3p. For western blot assays, coding region of PB1, PB2, PA and NP were amplified by PCR and cloned into pcDNA3.1(+) (Invitrogen). For ease of detection, flag tag was added to the 3′ primer, generating pcDNA3.1-flag-PB2, pcDNA3.1-flag-PB1, pcDNA3.1-flag-PA and pcDNA3.1-flag-NP. In order to further confirm the binding between miR-188-3p and PB2, the nucleotide sequence of putative binding sites in the pGL3-PB2–188-3p was mutant by overlap PCR. The mutant fragment was cloned into pGL3-cm to generate pGL3-mut-PB2–188-3p.

### Luciferase assay

HEK-293 T cells were seeded in 24-well plates and co-transfected with 200 ng of pGL3, 10 ng of pRL-TK (*Renilla*, Promega) and 60 nM miRNA mimics (Genepharma, Shanghai, China) by using Lipofectamine™ 2000 (Invitrogen). The scrambled miRNA was used as negative control. To ensure consistent transfection efficiency, we also added a control group that transfected FAM-labeled single-stranded negative control miRNA mimics in every experiment. The transfection efficiency was assessed by the fluorescent percentage at 24 h post transfection. Only experiments with transfection efficiency more than 70% were considered for further analysis. Forty-eight hours after transfection, cells were lysed in 100 μL of passive lysis buffer according to the Dual-Luciferase reporter assay protocol (Promega). After 10 min, the supernatants were collected by centrifugation at 12,000×*g* for 30s, and luciferase activity was measured by using the Dual-Luciferase reporter assay systems (Promega) on the Luminometer TD-20/20 (Turner Designs). The relative luciferase expression equals the expression of firefly luciferase (pGL3) divided by the expression of *Renilla* luciferase (pRL-TK). All experiments were repeated at least three times.

### Eukaryotic expression assay

To determine whether miRNA could repress the expression of target viral protein. HEK-293 T cells were plated in 12-well plates. When the cells reached a confluence of 50 to 60%, they were co-transfected with viral protein expression vectors (1.5 μg) and miRNA (60 nM). After 48 h, the cells were collected and analyzed by a Western blot assay and real-time PCR.

### Western blot

Total protein extracts of transfected cells were prepared for Western blot analysis in lysis buffer consisting of 150 mM NaCl, 1% NP40, 0.5% sodium deoxycholate, 0.1% SDS and 50 mM Tris-HCl pH 8.0 supplemented with a mixture of protease inhibitor (Roche). For immunoblotting, protein exacts were separated in 10% SDS-PAGE and transferred to PVDF membranes (Amersham Bioscience). Membranes were then incubated at room temperature for 1 h in a purified primary antibody (Sigma-Aldrich) at a 1:1000 dilution in 5% skim milk. After three washes with Tris-buffered saline containing 0.05% Triton X-100 (TBST), the membranes were incubated for 1 h at room temperature with the appropriate horseradish peroxidase-conjugated secondary antibody (Santa Cruz) at a 1:5000 dilution in 5% skim milk. Protein bands were visualized using the X-ray film, developing solution and fixing solution (Kodak) in darkroom. β-actin was used as a loading control.

### Real-time PCR analysis

Total RNA of transfected cells was prepared, and 2 μg of total RNA was reverse transcribed into cDNA using the PrimeScript™ 1st Strand cDNA Synthesis Kit (Takara) according to the manufacturer’s protocol. Quantitative real-time PCR was performed in triplicate on an ABI 7500 Real-Time PCR system using SYBR Green Master Mix (Takara). The mRNA levels were normalized to the expression of the housekeeping gene *β-actin*.

### Analysis of miRNAs on virus replication

To determine the effects of miRNAs on the replication of Influenza A virus, A549 cells were seeded in 12-well plates. When the cells reached a confluence of 80%, miRNA mimics (60 nM) were transfection in cells. After 6 h, the cells were infected with influenza A virus at a multiplicity of infection (MOI) of 0.01. At 0, 12, 24, 36, 48, 60 h post infection, supernatants were collected to measure 50% Tissue Culture Infective Dose (TCID_50_) in MDCK cells. At 48 h post infection, infected cells were harvested for total protein extraction and total RNA preparation.

### Virus infection

Influenza virus was diluted with RPMI 1640 media. A549 cells were washed with phosphate-buffered saline (PBS) three times and infected with influenza virus at a multiplicity of infection (MOI) of 0.01 for 1 h at 37 °C, 5% CO_2_ incubator. After incubation, the cells were washed with PBS three times, and cultured with RPMI 1640 containing 0.2% bovine serum albumin (BSA) (GIBCO) and 0.2 μg/mL TPCK Trypsin.

### Statistics analysis

Statistically significant differences were determined using one-way analysis of variance (ANOVA) with GraphPad Prism 5.0 software (San Diego, CA, USA). All of the assays were run in triplicate and are representative of at least 3 separate experiments. *P*-values less than 0.05 indicated significant differences.

## Results

### Screening of miRNAs that broad-spectrum targeting influenza A virus

**Fig. 1:**
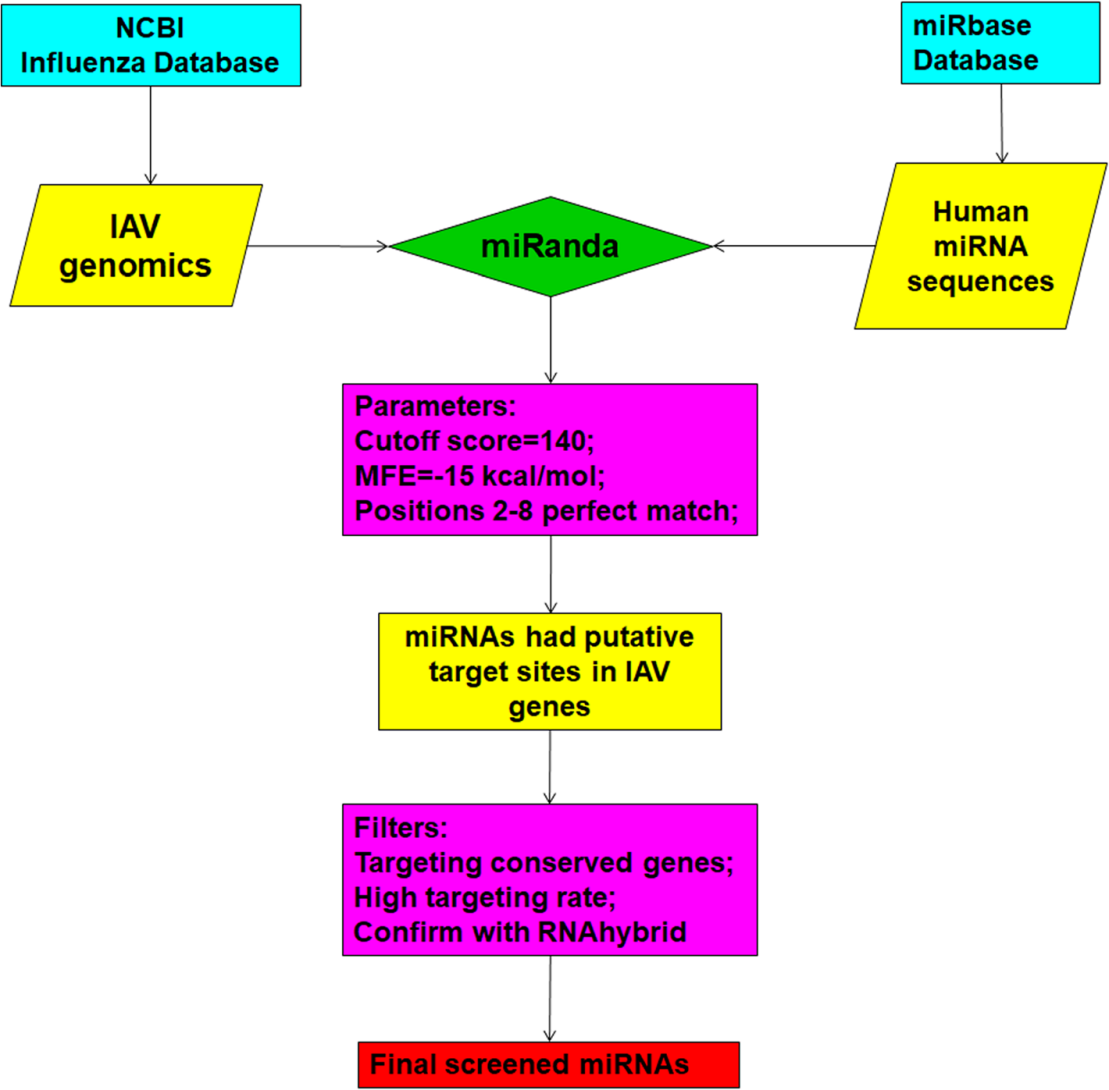
A systematic flowchart for screening miRNAs that broad-spectrum targeting Influenza A virus. All the data, software or databases are described in materials and methods

**Fig. 2:**
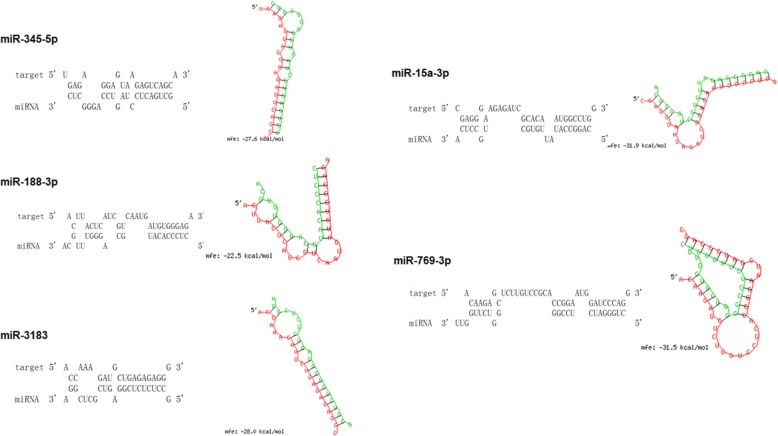
Binding between miRNAs and potential binding sites predicted by miRanda was confirmed by RNAhybrid

It is well known that IAV is characterized by pronounced genetic variation. To predict of miRNAs that broad-spectrum targeting human IAV, we first extracted all the sequence of IAV that can infected human from NCBI influenza virus Resource, 28,124 records in total. Human mature miRNAs sequence was downloaded from miRBase database, 2656 records in total. Then miRanda software was used to predict miRNA targets with the parameters mentioned in materials and methods. Figure 1 depicts the flowchart in the present study. miRNAs targeting polymerase gene (PB2, PB1 and PA) and nucleoprotein (NP) gene were considered for further study, because the four genes were relatively conservative and important to virus replication. Targeting rate of each miRNA was defined as the number of IAV strains that can be potentially targeted dividing by the total number of strains. Five miRNAs with high targeting rate were chosen for further research (Table 1). miR-345-5p and miR-188-3p potentially bind to PB2 gene with targeting rate of 95.31 and 99.96%, respectively. miR-3183 potentially bind to PB1 gene with targeting rate 92.90%. miR-15a-3p potentially bind to PA gene with targeting rate 94.58%. miR-769-3p potentially bind to NP gene with targeting rate 97.24%. The typical binding information was shown in Table 2. The putative binding sites were further verified using the RNAHybrid programs (Fig. 2). The results of this search suggested that it may exist cellular miRNAs broad-spectrum targeting IAV strains and may be developed to a universal antiviral therapeutic drug.

**Table 1:** Five miRNAs were selected for further research

Cellular miRNAs	Target	Targeting rate	Targeting rate of subtype											
			H1N1	H1N2	H2N2	H3N2	H5N1	H5N6	H7N3	H7N4	H7N7	H7N9	H9N2	H10N8
miR-345-5p	PB2	26,805/28124 95.31%	11,731/11821 99.24%	26/27 96.30%	80/81 98.77%	14,907/15773 94.51%	46/66 69.70%	1/4 25%	2/2 100%	1/1 100%	11/11 100%	0/325 0%	0/9 0%	0/4 0%
miR-188-3p	PB2	28,114/28124 99.96%	11,814/11821 99.94%	27/27 100%	81/81 100%	15,770/15773 99.98%	66/66 100%	4/4 100%	2/2 100%	1/1 100%	11/11 100%	325/325 100%	9/9 100%	4/4 100%
miR-3183	PB1	26,126/28124 92.90%	10,577/11821 89.48%	26/27 96.30%	81/81 100%	15,441/15773 97.9%	0/66 0%	0/4 0%	0/2 0%	1/1 100%	0/11 0%	0/325 0%	0/9 0%	0/4 0%
miR-15a-3p	PA	26,599/28124 94.58%	10,455/11821 88.44%	26/27 96.30%	79/81 97.53%	15,646/15773 99.19%	41/66 62.12%	2/4 50%	2/2 100%	1/1 100%	11/11 100%	323/325 99.38%	9/9 100%	4/4 100%
miR-769-3p	NP	27,347/28124 97.24%	11,716/11821 99.11%	27/27 100%	80/81 98.77%	15,516/15773 98.37%	3/66 4.55%	0/4 0%	1/2 50%	0/1 0%	0/11 0%	0/325 0%	4/9 44.44%	0/4 0%

Targeting rate was defined as the number of IAV strains that can be potentially targeted by miRNA dividing by the total number of strains

**Table 2:** A computational analysis of IAV genome yielded five sites are targets for these five miRNAs

Cellular miRNA	Target Gene (GeneBank entry) and Sites(nt)	Mfe (kcal/mol)	miRanda score	miRNA-mRNA pairing
miR-345-5p	PB2: CY009619 1437–1458	−22.96	159	
miR-188-3p	PB2: CY009619 1610–1629	−21.34	146	
miR-3183	PB1: CY009618 682–703	−24.79	160	
miR-15a-3p	PA: AJ238020 268–287	−25.34	157	
miR-769-3p	NP: AJ238021 505–530	−28.13	151	

The seed region of miRNAs is underlined. Nucleotide numbering is refered to A/FM/1/47(H1N1)

### miRNAs effectively inhibit luciferase expression according to luciferase assay

**Fig. 3:**
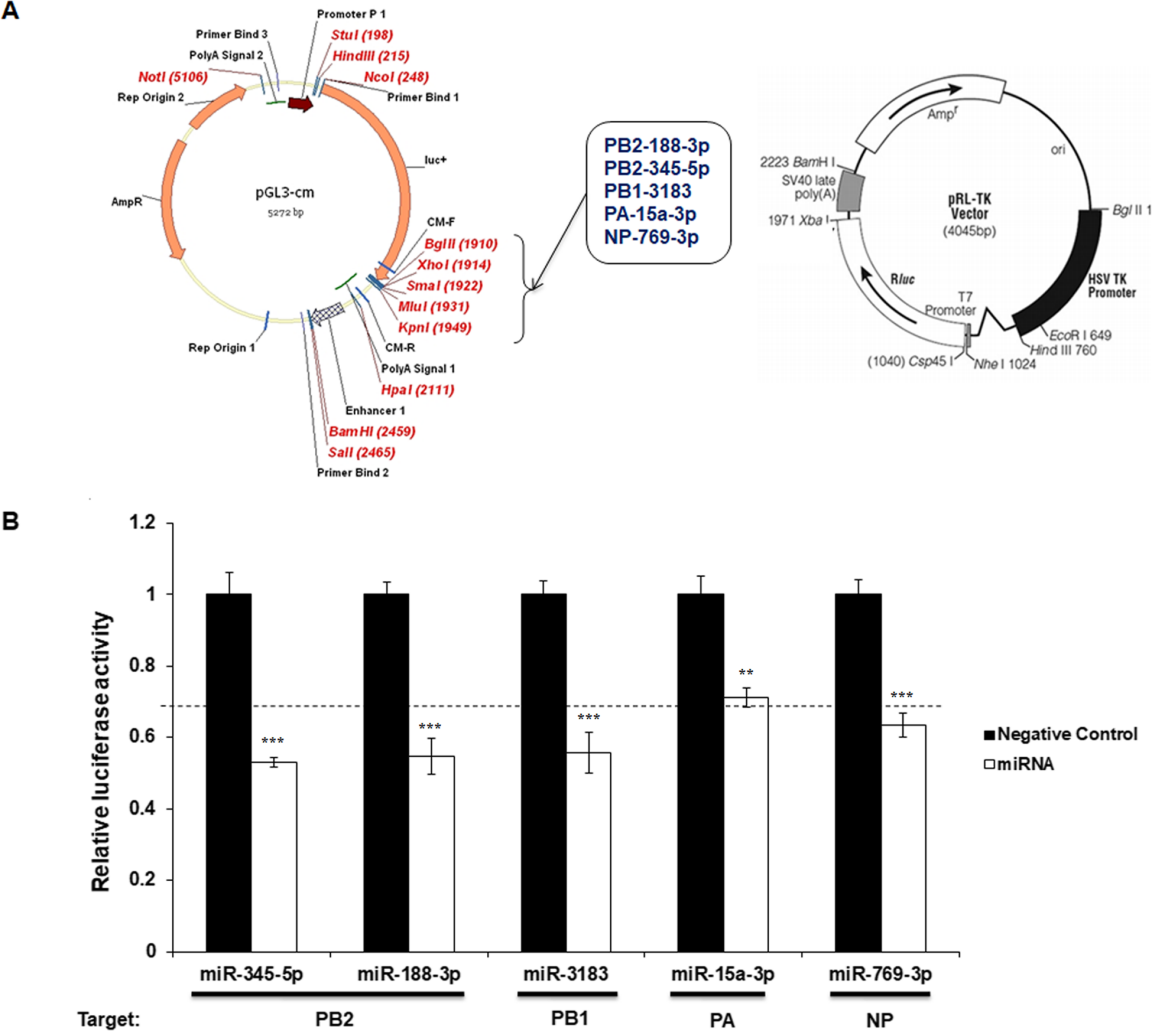
Four miRNAs may bind to the potential target sites. **a** Circular map of the pGL3-cm vector. The potential target sites of these 5 miRNAs were inserted into the Bgl II and Kpn I sites. pRL-TK vector was cotransfected to keep the balance of each group. **b** The relative luciferase activity from pGL3-cm reporter vector and pRL-TK vector. The relative luciferase activity of negative control miRNA transfected group was normalized to 1. The miRNAs which downregulated luciferase expressions by 30% or more, compared to the negative control group, were selected for further research. * (*P* < 0.05), **(*P* < 0.01), ***(*P* < 0.001), results were significantly different from negative control miRNA group

In order to determine whether predicted miRNAs target potential sites in IAV genome, the potential target sites of miRNAs were cloned into reporter vector pGL3-cm (Fig. 3a). HEK-293 T cells were cotransfected with reporter vector, control vector pRL-TK, miRNA mimics or the scramble control. As shown in Fig. 3b, miR-188-3p and miR-345-5p reduced the PB2 luciferase activity by 45.3 and 47.0%, respectively. miR-3183 reduced the PB1 luciferase activity by 44.3%, miR-15a-3p reduced the PA luciferase activity by 28.8%. miR-769-3p reduced the NP luciferase activity by 36.5%. We set 30.0% downregulation or more as cut-off, so miR-188-3p, miR-345-5p, miR-3183 and miR-769-3p were selected for further research.

### miRNAs downregulate the expression of corresponding viral protein and mRNA

**Fig. 4:**
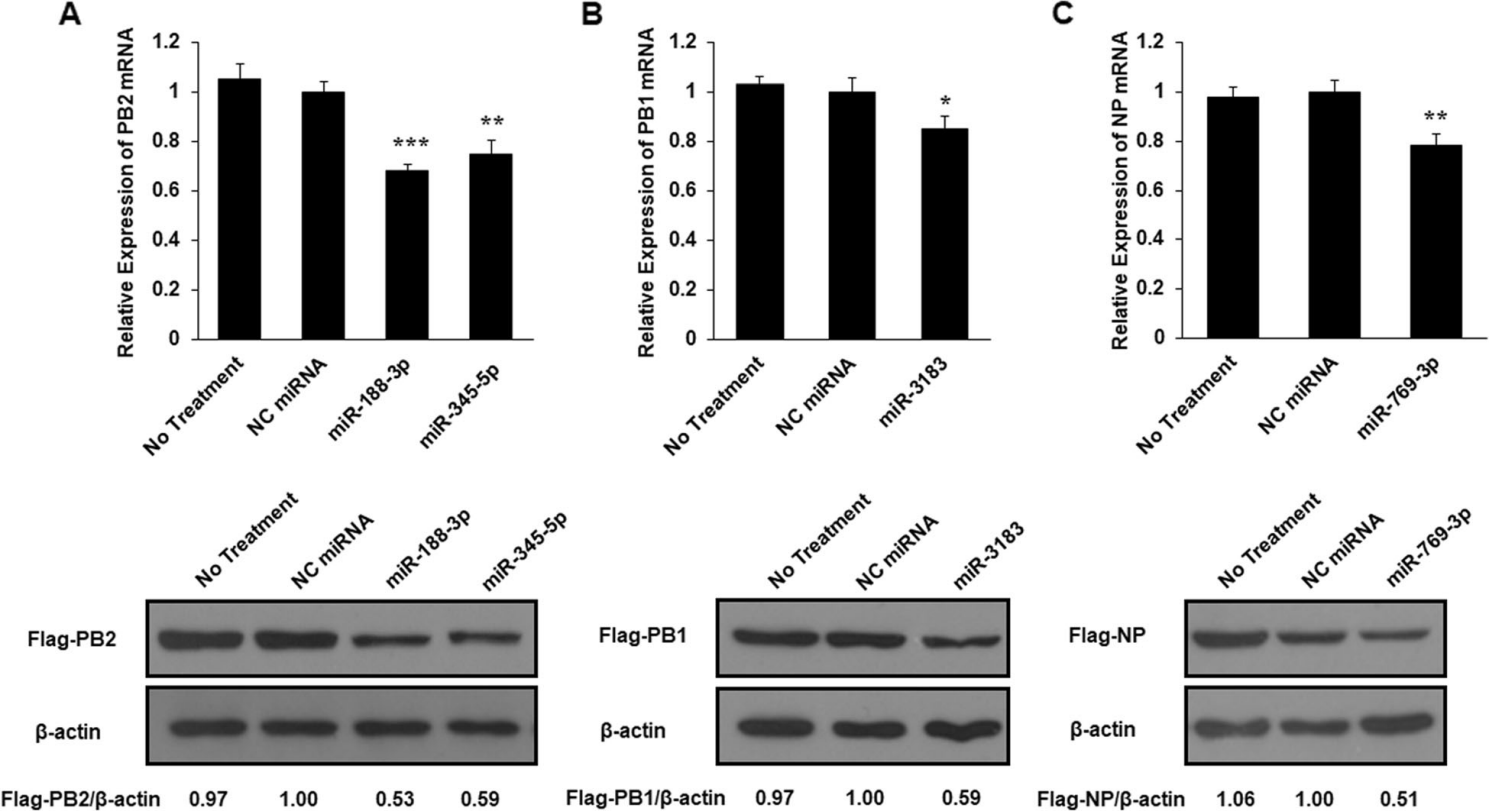
Four miRNAs downregulate expression of IAV mRNA and protein. QPCR (top) and Western bloting (bottom) was used to detect the effects of miR-345, miR-188-3p on PB2 expression (**a**), miR-3183 on PB1 expression (**b**) and miR-769-3p on NP expression (**c**). Densitometry depicting the ratio of Flag-PB2, Flag-PB1 or Flag-NP to β-actin for equal protein samples is indicated. * (*P* < 0.05), **(*P* < 0.01), ***(*P* < 0.001), results were significantly different from NC miRNA group

To further investigate whether miRNAs could regulate the expression of corresponding viral protein, we constructed pcDNA3.1-PB2, pcDNA3.1-PB1 and pcDNA3.1-NP vectors carrying CDS of PB2, PB1 and NP, respectively. HEK-293 T cells were contransfected with pcDNA3.1, miRNA mimics or the scramble control. As shown in Fig. 4 (bottom), viral protein expression was significantly downregulated when transfecting miRNA mimics, as compared with control miRNA or no treatment group, indicating that the four miRNAs could effectively inhibit the expression of viral protein. In addition, we utilized real-time PCR to detect the level of viral mRNA in HEK-293 T cells overexpressing miRNAs. The results showed that viral mRNA was significant reduced compared to the negative control group (Fig. 4 top), indicating that the four miRNAs downregulated viral gene expression at both mRNA and protein levels in HEK-293 T cells.

### miRNAs overexpression suppresses the replication of influenza A virus in A549 cells

**Fig. 5:**
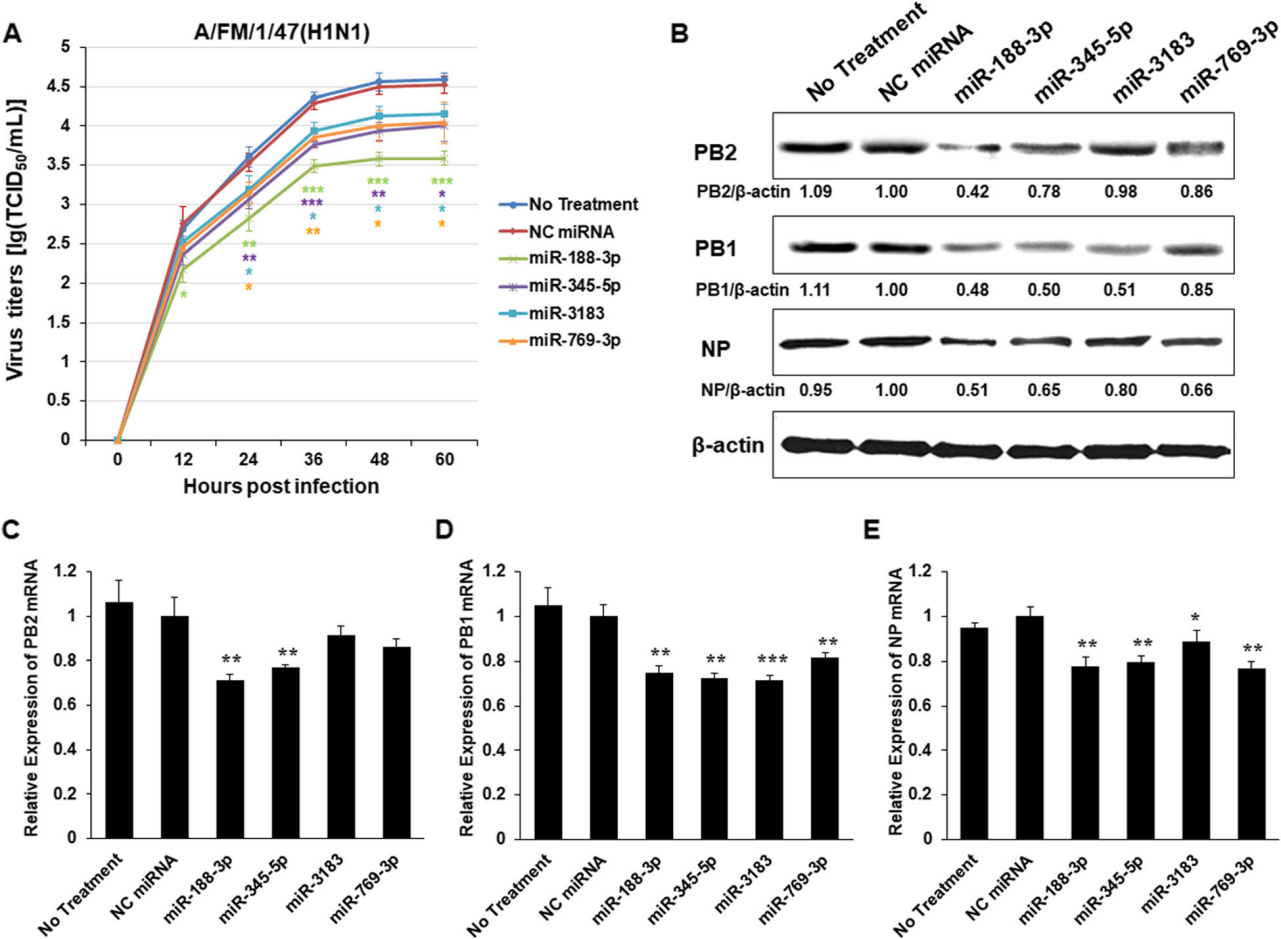
miRNAs inhibits the replication of H1N1 influenza A virus in A549 cells. **a** TCID_50_ values were used to assess the effects of miRNAs on replication of A/FM/1/47(H1N1) in A549 cells. Data were determined in triplicate at 0, 12, 24, 36, 48 and 60 h post infection (hpi). **b** Western blotting was used to detect the effects of miRNAs on PB2, PB1 and NP expression in A549 cells at 48 hpi. Densitometry depicting the ratio of PB2, PB1 or NP to β-actin for equal protein samples is indicated. **c**-**e** Real-time PCR was used to detect the expression level of the PB2 (**c**), PB1 (**d**) and NP (**e**) mRNA in A549 cells overexpressing miRNAs at 48 hpi. * (*P* < 0.05), **(*P* < 0.01), ***(*P* < 0.001), results were significantly different from NC miRNA group

Since miR-188-3p, miR-345, miR-3183 and miR-769-3p can downregulate the expression of viral protein, we then investigated whether overexpression of these miRNAs affect influenza A virus replication. A549 cells were tranfected with miRNA mimics or the scramble control, followed by infection with FM47 at MOI = 0.01. Supernatant was collected at 0, 12, 24, 36, 48 and 60 h post-infection (hpi) and the titers was determined by TCID_50_. As showed in Fig. 5a, miR-188-3p exhibited the most potent inhibitory activity on FM47 replication at 12, 24, 36, 48 and 60 hpi compared to the negative control, especially at 48 hpi, titers were 0.91 units (8.1 times) lower than that of negative control. The other three miRNAs, miR-345-5p, miR-3183 and miR-769-3p, could also significantly repress FM47 replication at 24, 36, 48 and 60 hpi. However, they were not as effective as miR-188-3p. In addition, we detected the expression of viral proteins and mRNAs in A549 cells overexpressing miRNAs at 48 hpi (Fig. 5b-e). The results were consistent with that of viral replication. All four miRNAs could inhibit the expression of viral proteins and mRNAs and miR-188-3p exhibited the most potent inhibitory activity. In summary, these result indicated that miR-188-3p could effectively inhibit FM47 replication.

In recent years, more and more highly pathogenic avian IAV crossed the interspecies barrier causing sporadic infections in humans with high fatality rate, such as H7N9 and H5N6. As shown in Table 3, the typical human-infected strains, A/Anhui/1/2013(H7N9), A/Shanghai/1/2013(H7N9), A/Shanghai/2/2013(H7N9) and A/Yunnan/0127/2015(H5N6) showed a higher binding strength with miR-188-3p than FM47. Because these human strains were not available in the present study, a quail H7N9 and a chicken H5N6 [35] with the similar binding strength were used to test the inhibitory effect of miR-188-3p. As shown in Additional file 1: Figure S1, miR-188-3p significantly lowered QA07 and CK918 titers by 1.09 units (12.3 times) and 1.02 units (10.5 times) at 48 hpi in A549 cells, indicating that miR-188-3p may also suppress the replication of emerging human-infected influenza A virus, such as H7N9 and H5N6 subtype.

**Table 3:** A computational analysis by miRanda suggested that miR-188-3p could also target H7N9 and H5N6

IAV strains	Mfe (kcal/mol)	miRanda Score	miRNA-mRNA pairing
A/Anhui/1/2013(H7N9) A/Shanghai/1/2013(H7N9) A/Shanghai/2/2013(H7N9)	−22.79	153	
A/quail/Hebei/CH06–07/2018(H7N9)	−19.50	153	
A/Changsha/1/2014(H5N6) A/Guangzhou/39715/2014(H5N6)	−16.38	147	
A/Yunnan/0127/2015(H5N6)	−22.79	153	
A/chicken/Hubei/XY918/2016(H5N6)	−22.23	157	
A/FM/1/47(H1N1)	−21.34	146	

The seed region of miRNAs is underlined

### miR-188-3p binds to the predicted site in the PB2 gene

**Fig. 6:**
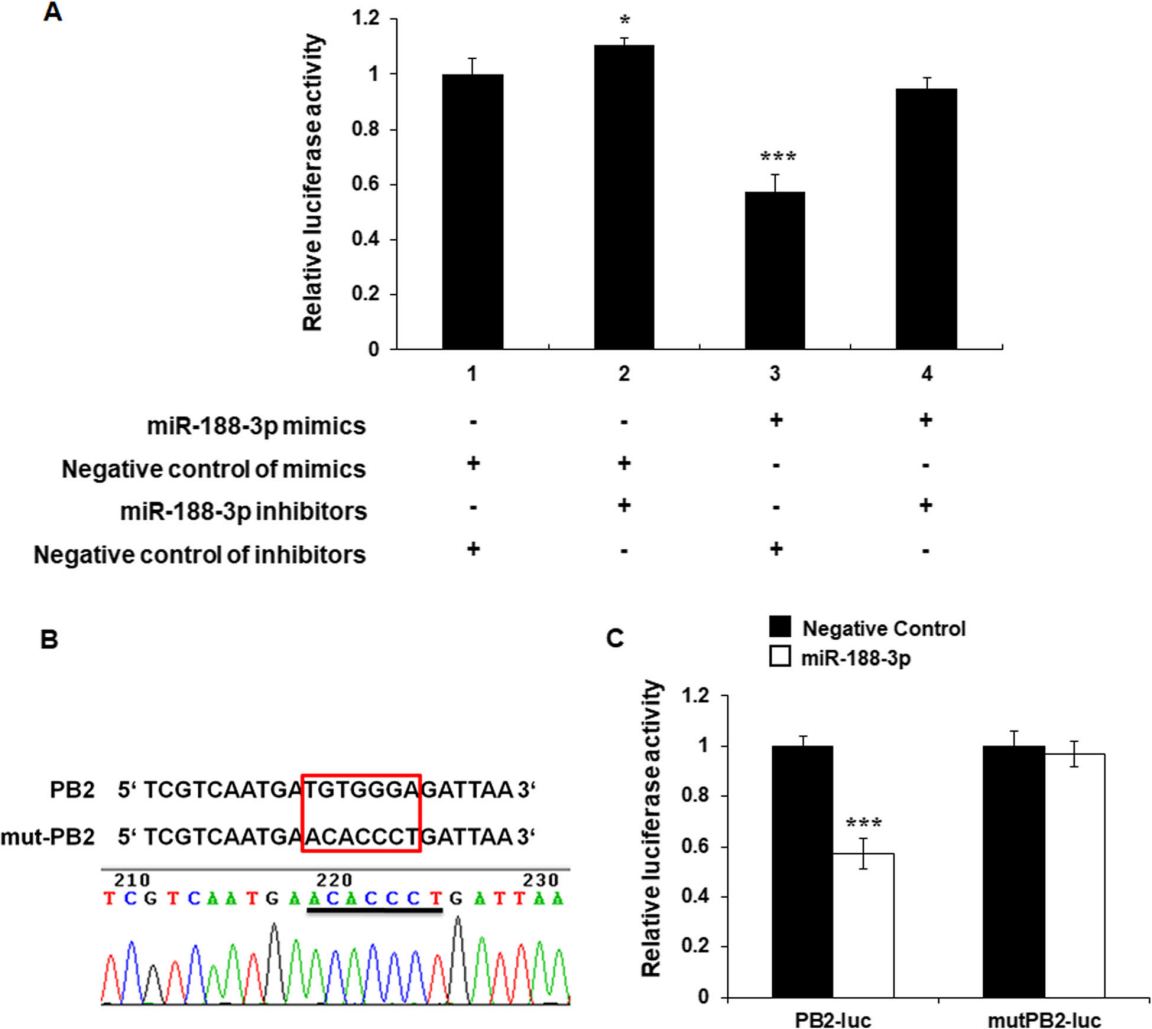
miR-188-3p inhibits the replication of IAV through targeting the potential binding sites on PB2. **a** specific miRNA inhibitors were contransfected into HEK-293 T cells with the miRNA mimics and dual-luciferase reporter vectors to further confirm the inhibitory effect of miR-188-3p on PB2 expression. **b** the potential binding sites of miR-188-3p seed region were mutant in the luciferase reporter vector. **c** Luciferase assay to analyze the importance of binding sites of miR-188-3p in the PB2 gene to inhibit the relative luciferase activity from pGL3-PB2–188-3p and pRL-TK-control vector. * (*P* < 0.05), **(*P* < 0.01), ***(*P* < 0.001), results were significantly different from negative control miRNA group

To further confirm the inhibitory effect of miR-188-3p on PB2 expression, specific miRNA inhibitors were contransfected into HEK-293 T cells with the miRNA mimics and dual-luciferase reporter vectors. As shown in Fig. 6a, the relative luciferase activity in transfected HEK-293 T cells with overexpressing negative miRNAs and miR-188-3p inhibitors (Fig. 6a, bar 2) was higher than that of control group (Fig. 6a, bar 1), which might cause by suppressing the endogenous miR-188-3p in HEK-293 T cells. Moreover, miR-188-3p inhibitors reversed the inhibitory effect of miR-188-3p on PB2 (Fig. 6a, bar 1,3,4). These results reversely validated that miR-188-3p could effectively inhibit PB2 expression.

Furthermore, we investigated whether miR-188-3p inhibit PB2 expression through binding to the predicted sites. The seed region binding site of miR-188-3p in PB2 gene was mutated in reporter vector (Fig. 6b). Treatment with miR-188-3p mimics reduced the reporter activity of the wild-type (WT), but not mutant (mut), luciferase reporter (Fig. 6c). These results suggested that miR-188-3p binds to the predicted site in the PB2 gene and the conservation of binding sites are very important for inhibitory activity.

## Discussion

IAV is well-known infectious disease that affects individuals of all ages in annual seasonal epidemic and uncontrollable periodic pandemic forms [36]. It is urgent to develop novel strategies for prophylaxis and treatment of the diseases. In recent years, cellular miRNAs that control IAV infection and replication has been well studied [37]. However, the broad-spectrum property, one of the key parameters to be developed as antiviral agents, had not been determined. In this study, we combined bioinformatics analysis and bio-experimental verification to screen cellular miRNAs that both effectively and universally inhibited the replication of IAV. With this approach, miR-188-3p was finally identified, which potentially targeted 99.96% of the human IAV strains and effectively suppressed IAV replication by binding to PB2 gene in A549 cells. Our work may therefore provide a comprehensive screening strategy of miRNA-based antiviral therapeutic interventions.

In this study, only miRNAs that potentially target the polymerase genes (PB2, PB1 and PA) or the nucleoprotein (NP) gene were considered. PB2, PB1 and PA together formed the RNA-dependent RNA polymerase protein complexes [38], and NP wrapped and protected the viral RNA. NP, viral RNA and the three polymerase proteins together formed ribonucleoprotein complexes (RNPs) during the replication process of IAV [39, 40]. The four proteins not only involved in the transcription and translation of the viral genes, but also played an important role in the nuclear export of viral RNA and protein and in the viral aggregation process [38]. In addition, the four genes were the relatively conservative segments of IAV.

The bioinformation prediction can help us to scale down time and cost by screening a few miRNAs which have surpassed the in-silico analysis for biological validation. However, high false positive rate existed in bioinformatics analysis. In this study, five broad-spectrum miRNAs were screened for further experimental verification. miR-15a-3p was excluded in the luciferase assay. All the other four miRNAs effectively repressed the expression of related target protein under plasmid transfection conditions. But, in the virus infection assays, miR-188-3p showed an obviously more effective virus inhibitory activity that the other three miRNAs. Therefore, a combination of bioinformation prediction and biological validation is an attractive strategy in enabling to propose new broad-spectrum therapeutic strategies to combat human IAVs in a very cost effective manner.

The reasons why the effect of miR-345-5p, miR-3183 and miR-769-3p was not as effective as that of miR-188-3p were complex. On the one hand, PB2 might be a more suitable anti-virus target than PB2, PB1 and NP. Because PB2 was not only responsible for viral gene expression and RNA genome replication, but also implicated as a host range determinant and pathogenicity factor [41–45]. Therefore, miR-188-3p might not only inhibit the replication of IAV but also reduce the virulence of IAV. As shown in Fig. 5a, the two miRNAs (miR-188-3p and miR-345) targeting PB2 exhibited a more inhibitory activity than the other two miRNAs (miR-3183 and miR-769-3p) targeting PB1 and NP, which might confirm this speculation. However, this still needed further verification. On the other hand, miR-188-3p exhibited a better inhibitory activity on the expression of PB2 proteins under both plasmid transfection conditions and virus infection conditions than miR-345-5p (Figs. 4 and 5b).

miRNA-based RNA interference has become a powerful new means to inhibit viral infection in a gene-specific manner, and diverse miRNA-based delivery system has been developed [46–49]. We believe that cellular natural miRNAs are more suitable for being developed as antiviral drugs than artificial siRNAs. Although their off-target effects cannot be totally avoided, endogenous miRNAs may have fewer side effects [29] and sometimes show a synergistic effect in the treatment of multiple diseases. Zhang et al. [50] found that miR-188-3p might be a new potential therapy for atherosclerosis by inhibiting macrophage proinflammatory response and oxidation. Pei et al. [51] found that miR-188-3p inhibited the cell proliferation and motility in breast cancer by targeting Transmembrane emp24 domain-containing protein 3 (TMED3). Pichler et al. [52] identified miR-188-3p as a novel prognostic marker and molecular factor involved in colorectal carcinogenesis. Our work expanded the insight into antiviral function of miR-188-3p.

In this study, three IAV strains was used to test the antiviral effectiveness of miR-188-3p. Although the binding pattern was representative, further studies should focus on verifying more IAV strains, especially that of different subtype and testing the protection effect of IAV challenge in animal models.

## Conclusions

In summary, this work for the first time developed a broad-spectrum anti-IAV miRNA screening strategy by using miRanda software, and found that miR-188-3p, potentially targeting 99.96% of human IAVs, could effectively repress IAV (H1N1, H5N6 and H7N9) replication in infected A549 cells by targeting PB2 mRNA. This strategy can be extended to any other virus researches, which provided valuable insight into the development of miRNA-based therapies against viral infection.

## Supplementary information

**Additional file 1: Figure S1.** miR-188-3p inhibits the replication of H7N9 and H5N6 influenza A virus in A549 cells. TCID_50_ values were used to assess the effects of miRNAs on replication of H7N9 (A) and H5N6 (B) influenza A virus in A549 cells. The strains used were A/quail/Hebei/CH06–07/2018(H7N9) and A/chicken/Hubei/XY918/2016(H5N6). Data were determined in triplicate at 0, 12, 24, 36, 48 and 60 h post infection. * (*P* < 0.05), **(*P* < 0.01), ***(*P* < 0.001), results were significantly different from NC miRNA group.

## Data Availability

All data generated or analysed during this study are included in this published article [and its supplementary information files].

## Abbreviations

IAV: Influenza A virus
miRNA: MicroRNA
MOI: Multiplicity of infection
NP: Nucleoprotein
NS: Nonstructural protein
PA: Polymerase acid protein
PB1: Polymerase Basic Protein-1
PB2: Polymerase Basic Protein-2
PBS: Phosphate-buffered saline
PCR: Polymerase Chain Reaction
RNAi: RNA interference
RNPs: Ribonucleoprotein complexes
TCID_50_: 50% Tissue Culture Infective Dose
TMED3: Transmembrane emp24 domain-containing protein 3
