# Supplementary material for: Identification of cellular microRNA miR-188-3p with broad-spectrum anti-influenza A virus activity
Source: Virol J. 2020 Jan 30;17:12. doi: 10.1186/s12985-020-1283-9 (PMC6993346; doi:10.1186/s12985-020-1283-9)

**Figure S1.** **miR-188-3p inhibits the replication of H7N9 and H5N6 influenza A virus in A549 cells.** TCID_50_ values were used to assess the effects of miRNAs on replication of H7N9 (A) and H5N6 (B) influenza A virus in A549 cells. The strains used were A/quail/Hebei/CH06-07/2018(H7N9) and A/chicken/Hubei/XY918/2016(H5N6). Data were determined in triplicate at 0, 12, 24, 36, 48 and 60 hours post infection. * (P<0.05), **(P<0.01), ***(P<0.001), results were significantly different from NC miRNA group.


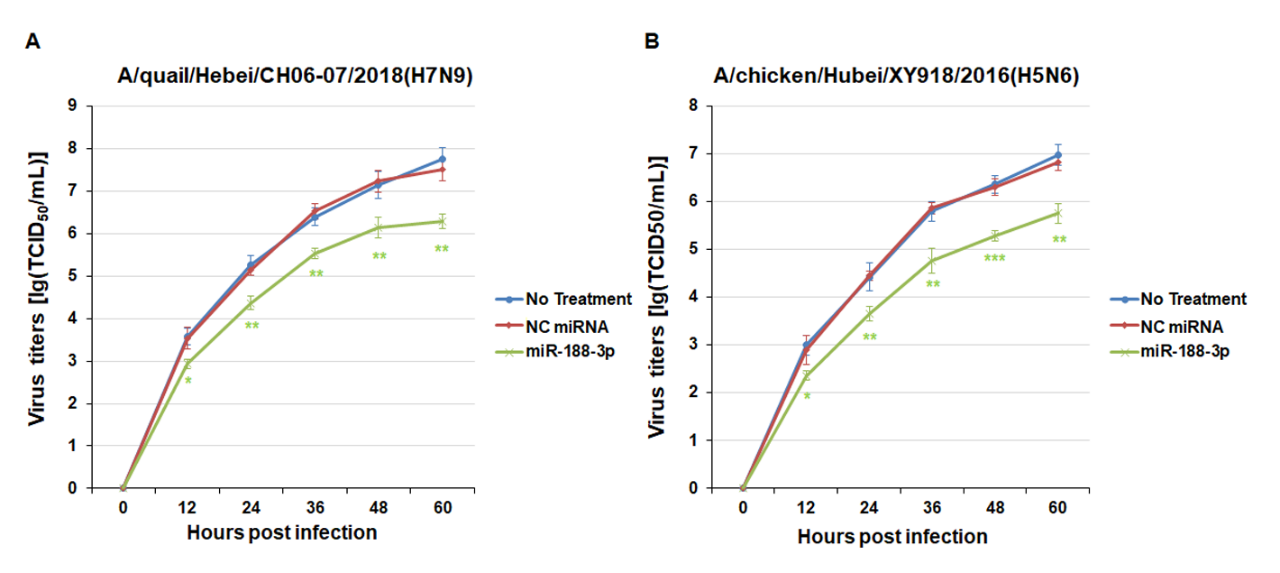

Supplement: Supplementary file 1 — Additional file 1: Figure S1. miR-188-3p inhibits the replication of H7N9 and H5N6 influenza A virus in A549 cells. TCID50 values were used to assess the effects of miRNAs on replication of H7N9 (A) and H5N6 (B) influenza A virus in A549 cells. The strains used were A/quail/Hebei/CH06–07/2018(H7N9) and A/chicken/Hubei/XY918/2016(H5N6). Data were determined in triplicate at 0, 12, 24, 36, 48 and 60 h post infection. * (P < 0.05), **(P < 0.01), ***(P < 0.001), results were significantly different from NC miRNA group. [file 12985_2020_1283_MOESM1_ESM.docx]
